# Selective DNA methylation in cancers controls collateral damage induced by large structural variations

**DOI:** 10.18632/oncotarget.10487

**Published:** 2016-07-08

**Authors:** Vakul Mohanty, Ogulsheker Akmamedova, Kakajan Komurov

**Affiliations:** ^1^ Systems Biology and Physiology Graduate Program, University of Cincinnati, OH, USA; ^2^ Graduate program in Mathematics, Fatih University, Istanbul, Turkey; ^3^ Division of Experimental Hematology and Cancer Biology, Cincinnati Children's Hospital Medical Center, OH, USA

**Keywords:** TCGA, computational cancer biology

## Abstract

Chromosomal instability is a hallmark of human cancers, and is characterized by large structural variations in the genome. Such large structural variations are expected to create intrinsic collateral stress due to gene dosage changes in many genes that are co-deleted or co-amplified in large chromosomal segments (*onco-passenger* genes). We show that the tumor-toxic effects of gene dosage changes of onco-passenger genes are compensated by the uncoupling of their copy number variations from their expression by means of selective DNA methylation. For example, collateral co-amplification of genes in tumor suppressor pathways, such as the TGF-β and inflammatory signaling pathways, are compensated by DNA hypermethylation to suppress their overexpression, while collateral deletion of pro-oncogenic genes are compensated by DNA hypomethylation to promote their expression from the single remaining allele. Our work reveals an important tumorigenic mechanism of regulation of toxic gene copy number imbalance in tumor cells arising from chromosomal instability, and suggests that targeting the DNA methylation machinery may prevent compensatory regulation of onco-passenger gene expression in chromosomally unstable cancers, and re-activate dormant tumor suppressor pathways for effective therapy.

## INTRODUCTION

Transformation to malignancy partly relies on the innovation of novel gene expression programs by tumor cells. A common mechanism employed by tumor cells to acquire novel gene expression programs is by structural genomic variations (SGVs). Many cancers are characterized by extensive genomic structural rearrangements that result in the gain or loss of chromosomal segments [1, 2]. Such structural rearrangements are thought to target specific “driver” genes, i.e. oncogenes or tumor suppressors, whose gain/loss of expression confers selective growth advantages to the transformed cell. However, structural rearrangements often involve large chromosomal segments [1], and therefore affect many other genes in the vicinity of the driver genes, leading to their copy number variations (CNVs). For example, loss of the *PTEN* tumor suppressor in many cancers is often accompanied by the loss of the entire chromosome 10 p-arm. Similarly, amplification of the *MYC* oncogene is usually associated with the amplification of the entire q-arm of chromosome 8, leading to the copy number gains of many hundreds of genes (Supplementary Figure S1). We call such genes “onco-passengers”, as their CNVs usually *accompany* the structural rearrangements involving the driver gene. Although the surge in the recent years, primarily owing to cancer genomics efforts such as The Cancer Genome Atlas (TCGA), have resulted in the identification of a large number of driver genes and processes in cancers [1–5], significantly less focus has been given to the onco-passenger genes. One notable study addressing the possible role of onco-passenger genes in cancers has found strong correlations of recurrent large structural changes with the density of oncogenes and tumor suppressor genes in the respective chromosomal segments [6, 7]. A more recent study has found that genes co-deleted with *TP53* on chromosome 17 in human cancers also have important roles in tumor suppression [8], suggesting that the onco-passenger genes may have previously unappreciated roles in tumor progression.

## RESULTS

### Onco-passenger gene expressions shape the tumor transcriptome

**Figure 1:**
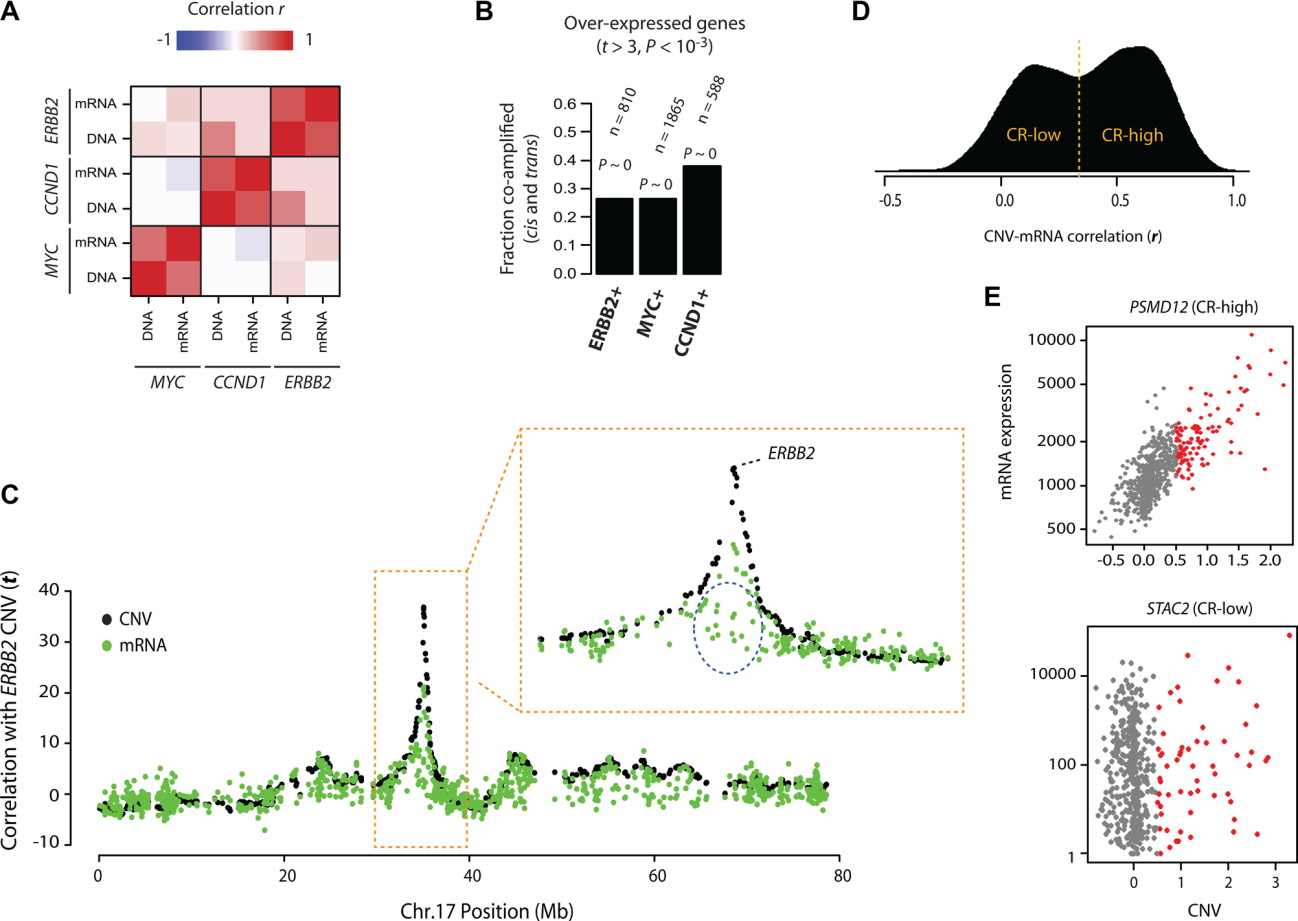
Patterns of onco-passenger gene expression (**A**) Correlations of co-amplification (DNA) and co-expression (mRNA) profiles of indicated oncogenes (*ERBB2*, *MYC* and *CCND1*) among each other (see text and Methods). High correlation indicates that genes that are co-expressed with the oncogene are also generally co-amplified. (**B**) Fractions of genes that are overexpressed with the indicated oncogene (at *P*-value of difference < 0.001) that are also co-amplified with the oncogene (in *cis* and *trans*). *P*-values reflect enrichment by hypergeometric distribution (*P* ∼ 0: machine zero). (**C**) Co-amplification (CNV) and co-expression (mRNA) profiles of genes on chromosome 17 with *ERBB2* CNVs. Y-axis shows correlation t-statistic; high “DNA” value indicates that the gene is co-amplified with *ERBB2*, while high “mRNA” value indicates it is co-expressed with *ERBB2* amplification (i.e. overexpressed when *ERBB2* is amplified). A portion of the chromosome 17 surrounding *ERBB2* gene is zoomed to show some genes (circled) that are not over-expressed, despite co-amplification with *ERBB2*. (**D**) Density plot of correlation (Pearson's *r*) of gene CNVs with their corresponding mRNA expression (i.e. how does change in CNV for a gene *X* correlate with the mRNA expression of gene *X*). CR-high and CR-low genes are defined by cutoff at the valley between the two distinct hills (usually at around *r* **=** 0.4). (**E**) CNV-mRNA scatter plots of a representative CR+ (top) and CR- (bottom) genes that are frequently co-amplified with *ERBB2* on chromosome 17. Fraction of samples with CNV gain is colored in red.

Changes in the expression of hundreds, or sometimes thousands, of co-amplified or co-deleted onco-passenger genes are expected to significantly affect the tumor transcriptome and have deleterious effects due to the gene copy number imbalances and collateral disruption of homeostatic processes [9]. First, we sought to assess the contribution of onco-passenger CNVs to the transcriptomic profiles of tumors associated with an oncogene amplification. For this purpose, we considered breast cancers, where amplifications of *ERBB2* (Chromosome 17), *CCND1* (Chromosome 11) and *MYC* (Chromosome 8) oncogenes are frequently observed (∼15%, 11% and 38%, respectively in the TCGA cohort). We measured the extent to which the transcriptomes of breast tumors with amplifications of *ERBB2*, *MYC* or *CCND1* oncogenes are due to the accompanying onco-passenger CNVs. To this end, we calculated two genome-wide metrics for each of the oncogenes above: 1) co-amplification profile: the correlation of its amplification with the CNV of every other gene in the genome (CNV-CNV correlation), and 2) co-expression profile: correlation of its amplification with the mRNA expression of every other gene in the genome (CNV-mRNA correlation). In the first, we measure the correlation of CNV of the oncogene of interest (i.e. *ERBB2, CCND1* or *MYC*) with the CNV of every other gene in the genome, and in the second, we measure the correlation of CNV of the oncogene with the mRNA levels of every other gene in the genome. The first metric captures the repertoire of co-amplified genes (onco-passengers) with the respective oncogenes, and the second metric captures the transcriptomic changes associated with each oncogene amplification. A correlation of the two metrics, therefore, is expected to reveal the extent to which the transcriptomics changes associated with an oncogene amplification (e.g. *ERBB2*) are due to the accompanying onco-passenger CNVs, as opposed to downstream pathway effects of the oncogene activation (e.g. PI3K/Akt or Ras/MAPK signaling). Intriguingly, we found that the two metrics have a very high correlation in each case (Figure 1A), suggesting that a significant portion of gene expression changes associated with *ERBB2-*, *MYC-* and *CCND1*-amplifying breast tumors are due to onco-passenger CNVs, which is also confirmed with an enrichment-based statistical test (Figure 1B). These observations indicate that the co-variations of onco-passenger gene copies are non-neutral, and have a substantial effect on the tumor transcriptomes.

### Many onco-passenger genes’ expressions are uncoupled from their CNVs

It is conceivable that onco-passenger genes associated with the amplifications of large chromosomal segments may contain genes that are deleterious to tumor viability. Similarly, deletions of large chromosomal segments may result in the co-deletion of genes with essential roles in tumor progression. In both of these cases, the expression of respective onco-passenger genes is expected to be actively uncoupled from their CNVs in tumors to allow for successful tumor progression. Indeed, for example in *ERBB2*-amplifying breast cancers, there are many genes within the *ERBB2* amplicon that are not co-expressed, despite high copy number co-amplification, with *ERBB2* CNV (Figure 1C). A similar pattern is observed with the onco-passengers of other oncogenes (e.g. *MYC*) and in other cancers as well (Supplementary Figure S2A). To test this phenomenon at a global level, we performed an analysis of correlations of onco-passenger CNVs with their respective mRNAs across a panel of cancers using TCGA datasets. In accordance with Figure 1C, we found that while many onco-passenger genes’ expressions in breast cancers changed in accordance with their copy number gains and losses (i.e. high CNV ∼ mRNA correlation), still many were insensitive to their CNVs (i.e. low CNV ∼ mRNA correlation, Figure 1D), a pattern that was repeated in other cancers (Supplementary Figure S2B). Moreover, the onco-passenger genes could be clearly classified into two groups based on the correlation of their expression changes with their CNVs (Figure 1D and Supplementary Figure S2B), with a distinct group of onco-passengers (CR-low: low CNV ∼ mRNA correlation) whose mRNA expression did not correlate with their copy number variations (see Figure 1E for examples of a CR-high and CR-low genes that are co-amplified with *ERBB2* on Chromosome 17). These observations suggest that tumors actively uncouple the expression of many onco-passenger genes from their CNVs, perhaps due to selective tumorigenic advantage.

### Onco-passenger gene expression reflects tumor advantage

**Figure 2:**
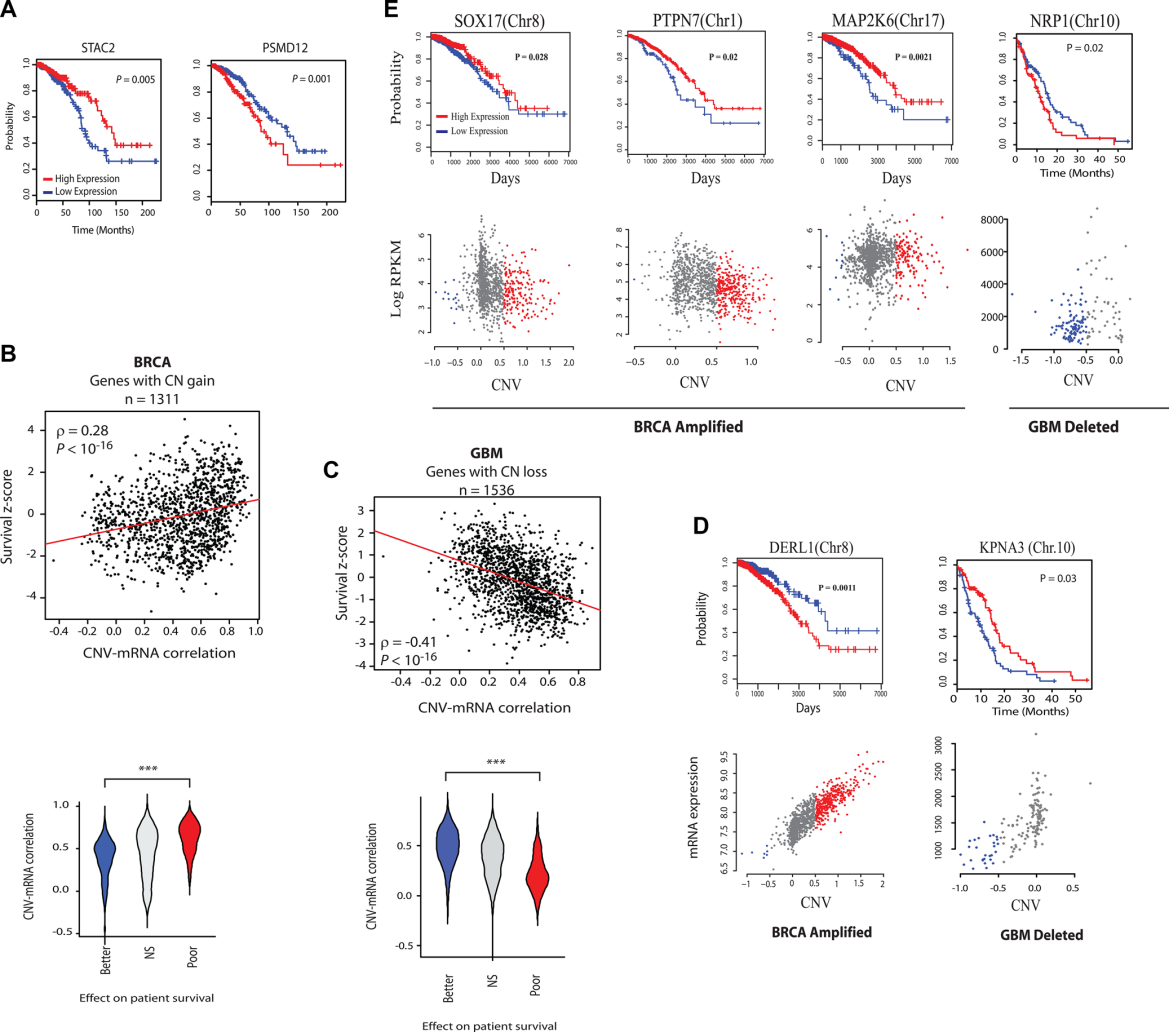
Analysis of the effect of CR-high and CR-low genes’ expression on clinical outcome (A) Kaplan-meier survival plots of a CR-low (STAC2) and CR-high (PSMD12) gene in breast cancer (BRCA). (**B**) Top: Scatter plots of CNV-mRNA correlation and COX regression z-scores of genes that are frequently amplified in BRCA. Red lines show the linear regression slope and ρ is Spearman's rank correlation coefficient. Bottom: Violin plots showing the distribution of CNV-mRNA correlation of genes that predict better (COX z-score < −2) and poor (COX z-score > 2) survival in BRCA, or have no strong predictive power (NS, |COX z-score| < 2). (**C**) Same as in (B), but with frequently deleted (CN loss) genes in GBM. Note the reversal of patterns from those in (B). (**D**) Survival and CNV-mRNA plots of some representative CR-high and (**E**) CR-low genes. Note reversal of survival impacts in CR-low genes with CN gain and loss. Chromosomal locations of genes are indicated.

Survival analysis of patients with high and low expression of the CR-high (PSMD12) and CR-low (STAC2) (see Figure 1E) genes that are co-amplified with *ERBB2* showed that while the expression of CR-high genes predicted poor survival, and hence more malignant cancer phenotype, expression of the CR-low gene had a better clinical prognosis, indicating less malignant cancer phenotype (Figure 2A). This suggests that CNV-mRNA correlation of onco-passenger genes may reflect tumorigenic advantage. To test this hypothesis, we measured the correlation of expression of every onco-passenger with clinical survival of patients in different cancers. Strong positive correlation of a gene's expression with poor survival reflects its possible role in conferring a malignant cancer phenotype, while a strong negative correlation would imply its role in suppressing tumor malignancy. Strikingly, we found that while amplified CR-high genes’ expression consistently correlates with poor survival, that of amplified CR-low genes predicts better clinical survival in many cases (Figure 2D–2ED and Supplementary Figure S3A), suggesting that amplified CR-low genes have tumor suppressor roles. This pattern was completely reversed in genes with copy number losses (Figure 2C), where the expression of CR-low genes predicted worse outcome, and hence more malignant phenotype, while CR-high genes predicted better clinical outcome (Figure 2C–2E), which was also observed in other, though not all, cancers (Supplementary Figure S3A). These observations suggest that collateral deleterious effects of gross structural changes in the tumor genome are at least partially compensated by active uncoupling of expressions of onco-passenger genes from their CNVs to achieve optimal tumor transcriptome.

### Onco-passenger gene expression is fine-tuned by DNA methylation

**Figure 3:**
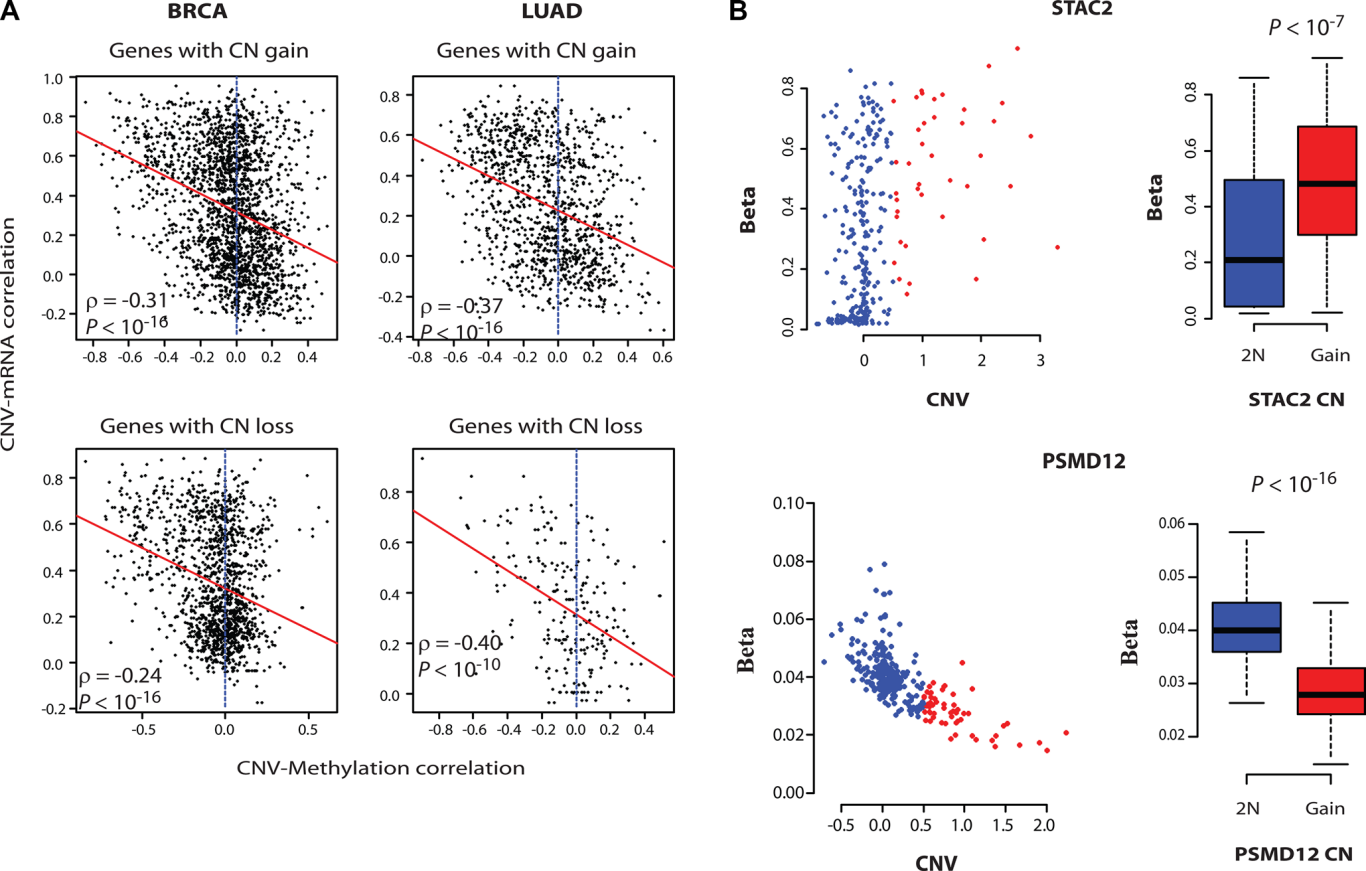
DNA methylation controls the expression of onco-passenger genes (**A**) For every gene, correlation of its CNV with its DNA methylation was measured. The scatter plots illustrate the relationship between CNV-mRNA and CNV-Methylation correlations, with red lines showing linear regression curve. (**B**) CNV-DNA methylation plots of an amplified CR-low (up) and CR-high (bottom) gene in BRCA. The boxplots to the right show quantification of changes in methylation beta values upon copy number gain.

To gain insight into the mechanisms of uncoupling of onco-passenger CNVs from their expression, we asked if the expression of amplified CR-low genes is actively suppressed, or the remaining copies of hemizygously deleted ones are actively induced, to compensate for their CNV. Epigenetic regulation through DNA methylation is a common mechanism of gene expression innovation in cancers [10]. Therefore, to test if DNA methylation plays a role in the uncoupling of CNVs of onco-passengers from their expression, we measured the correlation of DNA methylation of onco-passenger genes with their CNV. Strikingly, CNV-DNA methylation correlations showed a marked coherence with CNV-mRNA correlations of onco-passenger genes (Figure 3A). We found that most of amplified CR-high genes have loss of their DNA methylation, while many amplified CR-low genes have gains. Similarly, remaining copies of many hemizygously deleted CR-high genes are hyper-methylated, while many of hemizygously deleted CR-low genes are hypomethylated (Figure 3A and Supplementary Figure S3B) (see examples in Figure 3B). One possible explanation for this observation is that the DNA methylations of amplified CR-high genes are not maintained in the amplified extra copies, thereby manifesting as a hypomethylation phenotype. Similarly, the hemizygous deletion of CR-high genes might lead to the concentration of DNA methylation on the remaining allele, manifesting as a hypermethylation phenotype. However, CR-low genes, whose expressions are uncoupled from their CNVs, might maintain the DNA methylation of the amplified gene copies to suppress their over-expression, while deleted CR-low genes might lose DNA methylation in the remaining copy to maintain their expression. Nevertheless, these findings are intriguing, as they suggest that DNA methylation changes have a major role in the expression outcome of CN variations in cancers.

### Amplified CR-high onco-passengers code for oncogenic pathways, while CR-low onco-passengers code for tumor suppressor pathways

**Figure 4:**
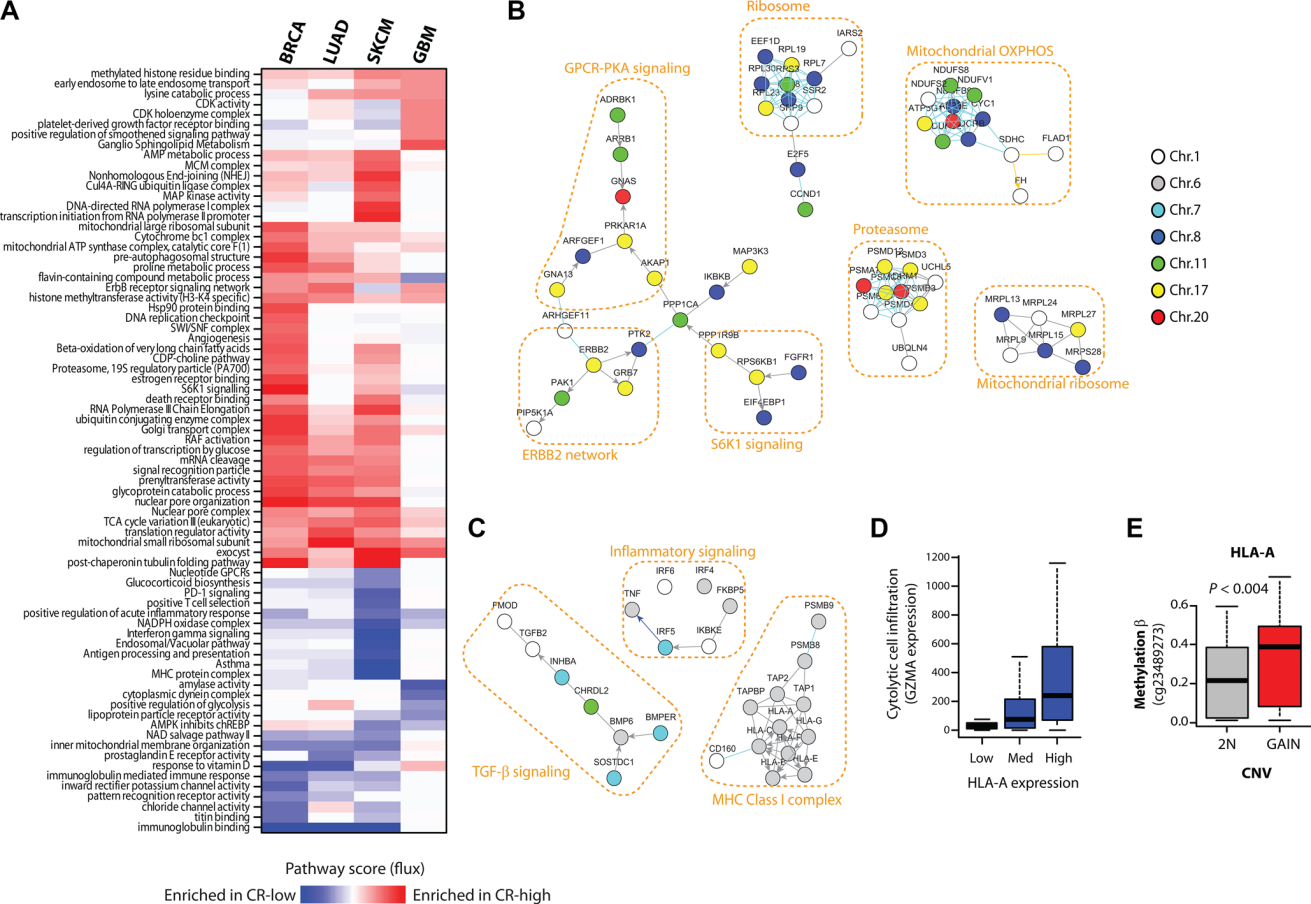
CR-low genes encode tumor suppressor pathways (**A**) Heatmap of pathway flux scores produced by NetWalk analyses of CNV-mRNA correlations of frequently amplified genes in BRCA, LUAD, SKCM and GBM. The interpretation of Pathway Flux scores are the same as in regular gene expression analyses, high positive score reflects the enrichment for CR-high genes, and a negative score reflects enrichment for CR- genes. (**B**) Network plot of some of the highest scoring (i.e. associated with CR-high genes) molecular interactions produced by NetWalk for BRCA. Node coloring reflects chromosomal locations. (**C**) Network plot of some most negative (i.e. associated with CR- genes) pathway scores in SKCM, see heatmap in (A). Node coloring is same as in (B). NetWalk analyses and network plots were produced in NetWalker [9]. (**D**) Boxplot of expression of GZMA in SKCM samples with low (< 30%-ile), medium (< 60%-ile) and high (> 60%-ile) expression of HLA-A. GZMA expression reflects infiltration by cytolytic cells, such as Natural Killer and CD8+ T cells. (**E**) DNA methylation beta values (probe indicated) of HLA-A gene in samples with no copy number gain (2N) and with copy number gain.

To test if CR-high and CR-low genes in different cancers are commonly associated with pro- or anti-tumorigenic pathways, we performed a pathway-level analysis of CNV-RNA correlations of commonly amplified genes using our previously developed network-based data analysis method, NetWalk [11, 12], in breast (BRCA), brain (GBM), lung (LUAD) and skin (melanoma, SKCM) cancers (see Methods). A heatmap of pathway scores reflecting enrichment of respective pathways in amplified CR-high or CR-low genes shows many expected pathways among amplified CR-high genes, such as increased CDK activity pathway in GBM and increased MAP kinase pathway in SKCM (Figure 4A). Intriguingly, in BRCA, in addition to the oncogenic ERBB2 signaling, the most prominent pathways associated with CR-high genes were involved in different aspects of protein homeostasis, including signal transduction (S6K1 signaling), protein synthesis (Ribosome) and degradation (Proteasome), many of which were co-amplified with *ERBB2* on chromosome 17, with *CCND1* on chromosome 11 or with *MYC* on chromosome 8 (Figure 4B).

In addition, a large subnetwork of CR-high genes in BRCA was involved in G-protein coupled receptor (GPCR) signaling through G_s_ and Protein Kinase A (PKA) (see Figure 4B). It is important to note that *PNMT*, a CR-high gene that is found within the same amplicon and almost always co-amplified with *ERBB2* on Chromosome 17, encodes phenylethanolamine N-methyltransferase, a critical enzyme in the synthesis of catecholamines (http://www.ncbi.nlm.nih.gov/gene/5409). Catecholamines, such as epinephrine, signal through β-adrenergic receptors to activate the G_s_-PKA pathway, which further supports the hypothesis that GPCR-PKA signaling plays a major role in breast cancers.

Pathways associated with CR-low genes, on the other hand, were often involved in innate and adaptive immune signaling (see Figure 4A), especially in SKCM (Figure 4C), where adaptive anti-tumor immunity plays a major role in tumor suppression [13]. Copy number gains in chromosome 6p are common in melanomas [14], although the driver oncogene(s) within this region are not well-defined. Interestingly, this region also harbors onco-passenger genes involved in MHC class I antigen presentation (see Figure 4C), a crucial mechanism required for T-cells to detect foreign (mutated) antigens in transformed cells. Accordingly, expression of these genes strongly correlates with local immune cell infiltration, as assessed by the expression of cytolytic cell (Natural Killer and CD8+ T cell) marker granzyme A (GZMA) (Figure 4D). However, expressions of these genes are uncoupled from their copy number gains, at least partly due to hypermethylation of their promoters (Figure 4E), thereby avoiding immune cell infiltration and anti-tumor activity. Overall, our pathway analyses confirm pro- and anti-tumorigenic roles of amplified CR-high and CR-low genes, respectively, and shed light on the mechanisms of their role in tumor progression.

## DISCUSSION

Structural changes due to genomic instability in cancers are usually non-selective, and can result in copy number gains or losses of hundreds of genes [1, 2, 15]. The research efforts in cancer genomics have mainly focused on the driver genes, and less attention has been given to the onco-passengers that have undergone similar CNVs as the driver genes. Previously, we showed that the pathway expression landscape of breast cancers with *ERBB2* amplifications is largely driven by gene CNVs [16]. In this study, we confirm this finding at a broader scale, and show that onco-passenger gene expression has a significant contribution to the tumor transcriptomes. However, still many onco-passengers’ expressions are actively uncoupled from their CNVs to shield the deleterious effects of their CNVs on tumor survival. Indeed, we found that some amplified onco-passenger genes code for tumor suppressor pathways, such as TGF-β and inflammatory signaling, whose expression predicts less malignant disease and better patient survival. These genes are thus actively repressed upon their amplification, at least partly through promoter hyper-methylation, which avoids “collateral” activation of tumor suppressor pathways.

Methylation of CpG sites on DNA is a major mode of epigenetic control of gene expression, and its role in the suppression of tumor suppressor genes has been established [10]. Our results here show that DNA methylation has a role in fine-tuning the effects of gross SGVs, and that it cooperates with gene copy number changes in shaping the optimal tumor transcriptome. Thus, these observations further strengthen the rationale for targeting of DNA methylation in human cancers [17, 18], especially those with gross SGVs, which could lead to the re-activation of onco-passenger tumor suppressor genes.

## MATERIALS AND METHODS

### Datasets

All of the datasets were obtained from TCGA data portal. CNV of genes were obtained from the segmentation data of SNP 6.0 arrays by *CNTools* package for R. RNAseq V2 datasets were used for gene expression data (Normalized count data). For methylation data, the Infinium 27 k array data were used for all, except SKCM (where 27 k data were not available), datasets. For SKCM, Infinium 450 k data were used with the 27 k probes for consistency with other datasets. Datasets used and their sample sizes are shown in Supplementary Table S1.

### Computational analyses

In all analyses, genes with CNV gain were defined as those that had 90%-ile (among all patients) CNV (log ratio as measured in SNP6 array) of > 0.50, and those with CNV loss were defined as genes that had 10%-ile CNV of < −0.50. To exclude genes that are constitutively suppressed in a given cancer type, only genes that have expression > 30 normalized counts in at least 10% of the tumor samples were included in the analysis. Survival analyses were conducted with COX regression analysis using R package *survival* (coxph function).

### Pathway analyses

Pathway analyses were conducted in NetWalker [11] using the CNV-mRNA correlation values for NetWalk scoring. Briefly, CNV-mRNA correlation values, where CR-high genes were defined as those having correlation *r*-values of > 0.4, were transformed by substracting 0.4 (*f(x) = x – 0.4*), so that CR-high genes now have positive values and CR-low genes have negative values (for more intuitive separation of CR-high and CR-low genes/pathways). Then, the data were transformed by *f(x) = 2^x^* prior to NetWalk to make the data positive and centered around 1 (a requirement of NetWalk, see ref.^10^). NetWalk was run on the resultant values using default parameters in NetWalker to obtain Pathway Flux scores for each pathway. The heatmap in Figure 4A is a clustered heatmap of Pathway Flux scores from selected most positive (enriched in CR-high) and most negative (enriched in CR-low) scoring pathways.

## SUPPLEMENTARY FIGURES AND TABLE


