# Supplementary material for: Selective DNA methylation in cancers controls collateral damage induced by large structural variations
Source: Oncotarget. 2016 Jul 8;8(42):71385–92. doi: 10.18632/oncotarget.10487 (PMC5641056; doi:10.18632/oncotarget.10487)
Supplement: Supplementary file 1 [file oncotarget-08-71385-s001.pdf]

Selective DNA methylation in cancers controls collateral damage induced by large structural variations

Supplementary Materials

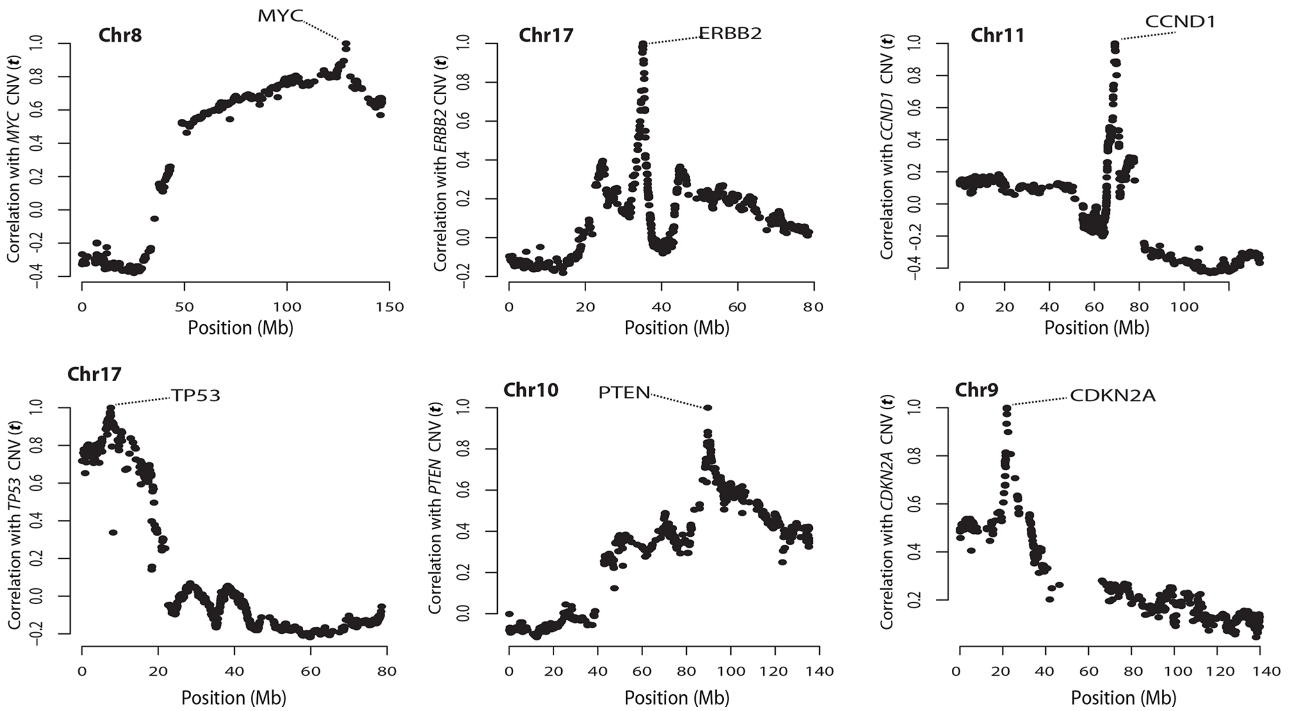

Supplementary Figure S1: Co-amplification and deletion patterns across the chromosome is depicted by plotting the spearman coefficient of the correlation between copy numbers of each gene with the corresponding driver gene (indicated in figure). The top three plots are examples of amplified driver genes (MYC, ERBB2 and CCND1), the bottom plots are examples of deleted driver genes (TP53,PTEN,CDKN2A).

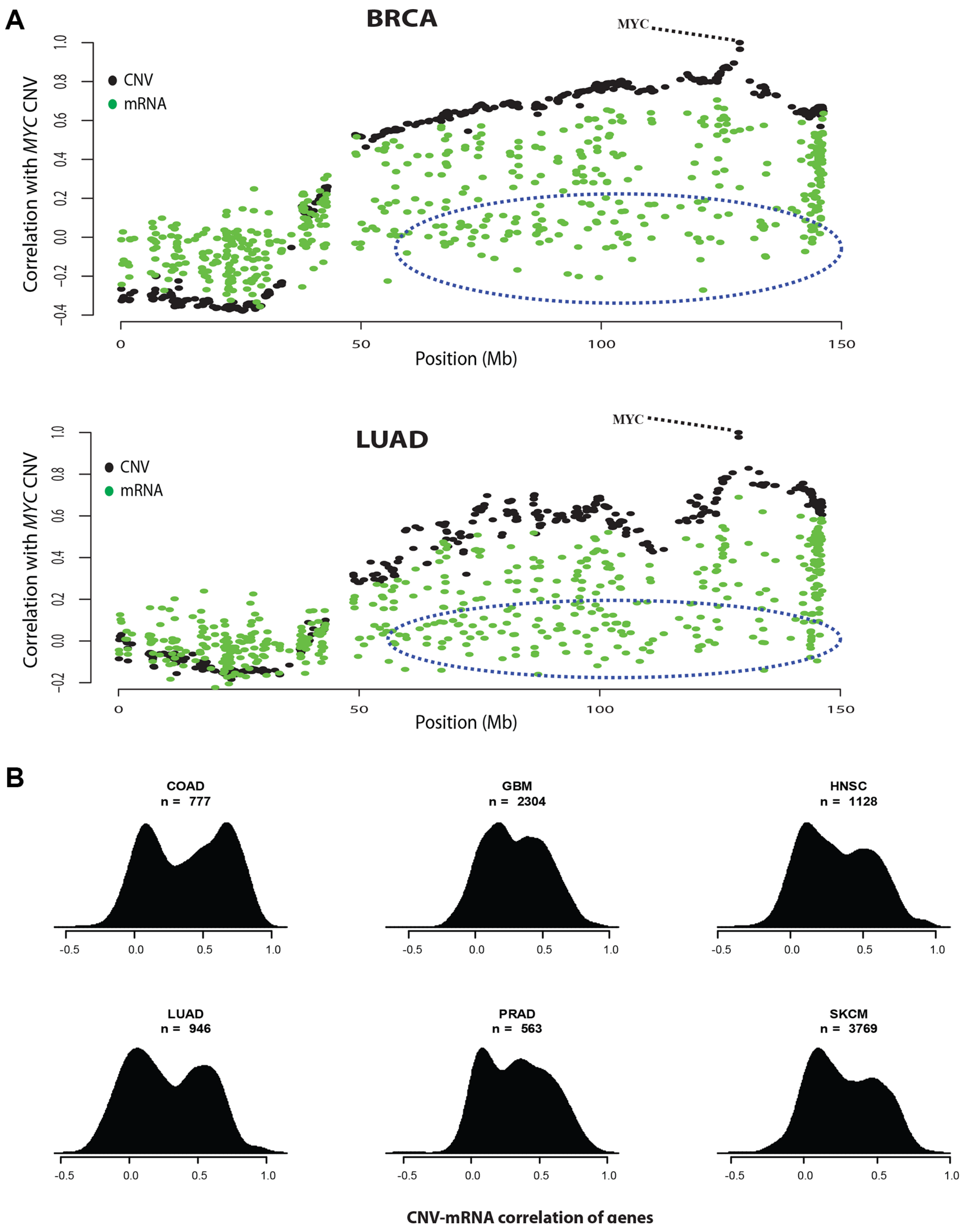

Supplementary Figure S2: (**A**) Spearman correlation between copy number (*black*) and mRNA expression (*green*) of each gene in chromosome 8 with the CNV of *MYC* in BRCA and LUAD were plotted against the chromosomal positions of genes. It shows large regions on chromosome 8 where genes are co-amplified with MYC but aren't co-expressed. (**B**) Density plots of CNV-mRNA correlations of genes with frequent copy number gains or losses in indicated cancers. Note bimodal patterns, suggesting the existence of a distinct population of CR- genes whose expression is uncoupled from their CNVs.

Supplementary Table S1: TCGA datasets used in the study and their sample sizes

	RNA-seq	CNV	Infinium 27k
**BRCA**	1095	1089	316
**COAD**	283	506	166
**GBM**	169	580	295
**HNSC**	522	526	-
**LUAD**	353	366	126
**PRAD**	498	497	-
**SKCM**	473	471	197

DNA methylation data were not used for HNSC and PRAD.

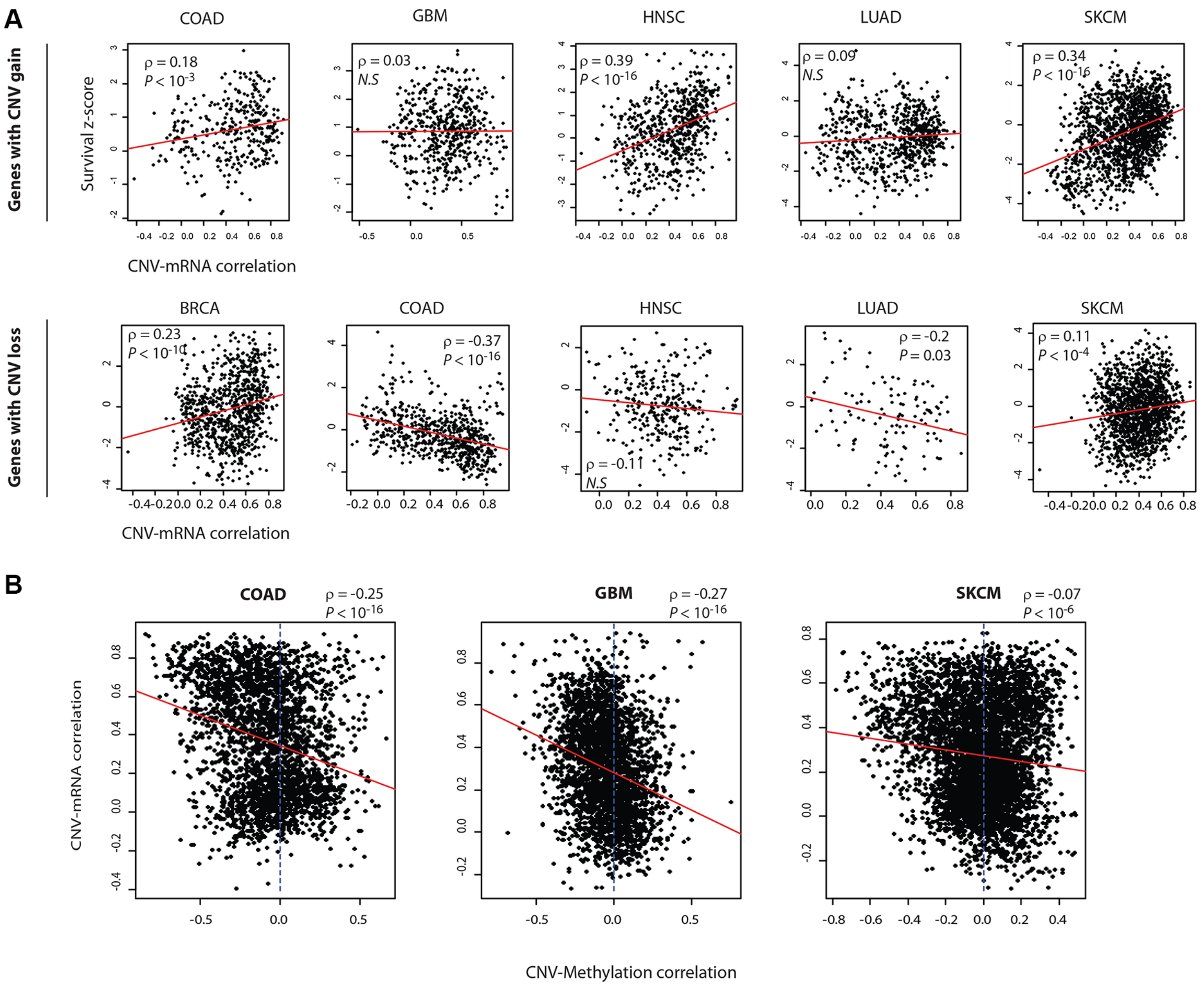

Supplementary Figure S3: (**A**) Scatter plots of CNV-mRNA and survival z-scores (COX regression) of genes with CN gain (top) or loss (bottom) in indicated cancers. Plots were done the same way as in Figure 2B and 2C. (**B**) Scatter plots of CNV-Methylation and CNV-mRNA correlations of genes with frequent gain or loss in the indicated cancers. Plots were done the same way as in Figure 3A.
